# A Systematic Review of Clinical Practice Guidelines for Neonatal Abstinence Syndrome

**DOI:** 10.3390/children10101685

**Published:** 2023-10-13

**Authors:** Zoe Wei, Yasmin Gilbert, Arabhi Thananjeyan, James Cope, Rachael L. Morton, Annie Li, Cecile T. Pham, Meredith Ward, Ju Lee Oei

**Affiliations:** 1 School of Women’s and Children’s Health, Faculty of Medicine, University of New South Wales, Sydney, NSW 2052, Australia; zoe.wei@student.unsw.edu.au (Z.W.);; 2 Prince of Wales Hospital, Barker Street, Randwick, Sydney, NSW 2031, Australia; 3 National Health and Medical Research Council Clinical Trials Centre, The University of Sydney, 92–94 Parramatta Road, Camperdown, Sydney, NSW 2006, Australia; 4 Department of Newborn Care, The Royal Hospital for Women, Barker Street, Randwick, Sydney, NSW 2031, Australia

**Keywords:** neonatal abstinence syndrome, neonatal opioid withdrawal syndrome, clinical practice guidelines, prenatal opioid exposure, development

## Abstract

Background: The prevalence of neonatal abstinence syndrome is increasing, but the number and quality of clinical practice guidelines available are unknown. This systematic review aimed to identify, appraise and evaluate clinical practice guidelines for neonatal abstinence syndrome. Methods: A systematic search of databases and the grey literature was conducted between 1 June and 1 July 2022. Full-text guidelines published by national or state-wide institutions were included. The recommendations from each guideline were extracted. The AGREE-II instrument was used to assess guideline quality. Sufficient-quality scores were defined as >60 and good-quality scores were >80 for each domain of AGREE-II. Results: A total of 1703 records were identified, and 22 guidelines from the United States, Australia, Canada and the United Kingdom, published between 2012 to 2021, were included. The quality scores were low, with median scores of 37/100 for stakeholder involvement, 33/100 for methodology, 34/100 for applicability and 0 for editorial independence. Scope and purpose scored 72/100, and presentation scored 85/100. Sixteen (73%) guidelines did not meet the cut-offs for clinical use. Conclusion: Many guidelines were of insufficient quality to guide clinical practice for neonatal abstinence syndrome. This emphasises the need for high-quality studies to inform clinical practice guidelines, improve care and reduce the risk of poor outcomes in these high-risk infants.

## 1. Introduction

Over recent decades, the use of prescription and illicit opioids by pregnant women has increased dramatically, corresponding with a rapid rise in the number of infants with neonatal abstinence syndrome (NAS) [1,2]. In the United States of America (USA), the prevalence of NAS increased from 4 to 6.3 per 1000 births from 2010 to 2020 [3]. Infants with NAS cost significantly more than non-NAS infants (USD $9200 versus USD $1300) due to perinatal complications such as low birth weight and respiratory distress, and prolonged hospitalisation for NAS treatment [2,3,4]. While variable, anywhere from 19–52% of substance-exposed infants have been reported to require pharmacological withdrawal treatment with replacement opioids [5]. American hospitals report average post-natal lengths of stay of nine days for NAS infants, compared to two to three days for otherwise healthy full-term infants [3]. 

NAS is a difficult condition to treat. The severity of an infant’s withdrawal is influenced by multiple factors, such as the type and dosage of maternal drug intake, gestational age and genetic susceptibility [6]. Assessments may be subjective, and there is no standardised, universally accepted pharmacological or non-pharmacological treatment regime for withdrawal treatment [6,7]. Clinicians can choose from an armamentarium of interventions, including various short- and long-acting opioids, anti-seizure medications, centrally acting antihypertensive agents and non-pharmacological methods, including acupuncture [8]. However, there is a paucity of good-quality evidence underpinning these common practices for NAS, and practice changes frequently, as has been acknowledged in the literature [6,7]. In a review of 53 articles published between 2007 and 2017, Wachman et al. (2018) found only five randomised controlled trials assessing pharmacological treatment with small (*n* = 26–131) sample sizes, and none examined long-term outcomes [9]. 

In general, best practice management is informed by clinical practice guidelines (CPGs), documents defined by the Institute of Medicine as “recommendations intended to optimise patient care that are informed by a systematic review of evidence and an assessment of the benefits and harms of alternative care options” [10]. CPGs have been shown to consistently decrease healthcare utilisation and costs, and to improve practice consistency and patient outcomes, including in NAS [11]. Hall et al. (2015) demonstrated that stringent protocols for pharmacological treatment and the weaning of NAS resulted in a significant overall reduction in length of stay from 32 to 24 days when compared to standard practice [11]. 

While there are clear benefits as to the use of CPGs when guiding the clinical management of NAS infants, the quality of existing CPGs is unknown. In this study, our aim was to identify and evaluate the quality of publicly available, contemporary CPGs for the diagnosis and management of NAS, and assess their suitability as guidance in routine clinical care. 

## 2. Methods

### 2.1. Search Strategy

**Figure 1:**
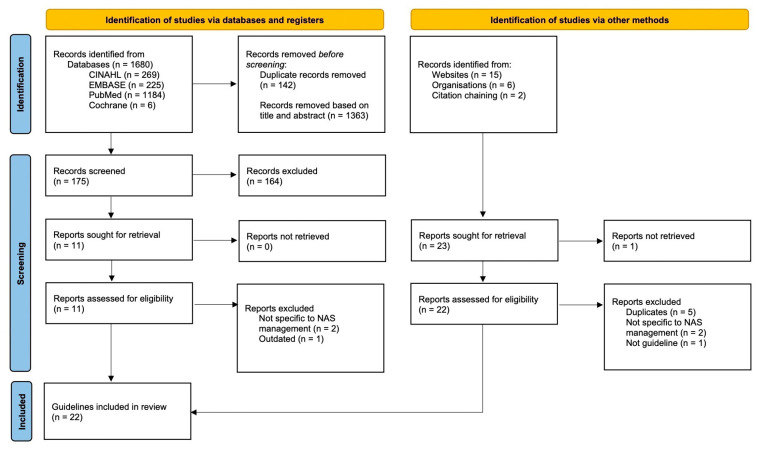
Flow chart of search and selection process. The flow chart was based on the 2020 PRISMA statement [12]. CINAHL: Cumulative Index to Nursing and Allied Health Literature; EMBASE: Excerpta Medica database; NAS: neonatal abstinence syndrome.

A systematic review was conducted in accordance with the Preferred Reporting Items for Systematic Review and Meta-Analyses (PRISMA) Statement [12]. Searched databases included Medline, Embase, Scopus, Translating Research Into Practice, the Guidelines International Network, the National Guideline Clearinghouse, the National Institute for Health and Care Excellence database and Google Scholar from data inception to 31st June 2022 (shown in Figure 1). Search terms included neonatal, abstinence, withdrawal, opioids and guidelines. We also searched reference lists of eligible review articles and contacted experts in the field for relevant articles. We did not apply language restrictions or limitations on publication status (i.e., guidelines not reported in peer-reviewed medical journals were included). 

### 2.2. Study Selection 

The guidelines were independently reviewed by five assessors (ZW, YG, CTP, JC, AL). Both published and unpublished guidelines at a national, state and organisational level were included. Guidelines without full text were excluded, as were editorials, conference papers and narrative reviews of NAS management. Guidelines that did not focus on the management of NAS in any aspect (i.e., the obstetric management of pregnant women using substances) were also excluded. In all cases, the original and most recently published guideline was sourced. For guidelines with more than one version, only the most recent version was included. The remaining guidelines were included if they focused on any aspect of the antenatal or postnatal management of NAS. 

### 2.3. Data Extraction 

Data were extracted independently by two reviewers (ZW, YG). Data extracted from relevant guidelines included:

1. Antenatal screening for neonates at risk;
2. Diagnosis of NAS;
3. Post-natal care of infants, including non-pharmacological and pharmacological management of NAS;
4. Follow-up.

The key characteristics and clinical recommendations in the guidelines are summarised and collated in Table 1 and Table 2. 

### 2.4. Quality Assessment of the Evidence Supporting the Guideline Recommendations

The methodological quality of all relevant guidelines was assessed using the Appraisal of Guidelines for Research and Evaluation (AGREE) II Instrument by five independent reviewers (ZW, YG, AT, JC, AL) [13]. This tool was recommended by Johnston et al. for methodological appraisal, and consists of 23 items organised within six domains [14]. These are followed by one global rating item, “Overall Assessment”, which was based on the reviewer’s judgement of the guideline, and accounted for the scores of each domain. Each domain captures a unique dimension of guideline quality: scope and purpose (three items), stakeholder involvement (three items), rigor of development (eight items), clarity of presentation (three items), applicability (four items) and editorial independence (two items). Each of the 23 items were considered to be equal in weight, and were scored on a seven-point scale from one (strongly disagree) to seven (strongly agree). A scaled quality score was calculated for each domain using a formula provided in the AGREE II user manual (considering the number of appraisers) [13]. 

Guidelines were also examined for quality cut-offs, central tendency and intraclass correlation coefficients (ICC). AGREE-II does not set cut-offs within the instrument, so these were based on previous paediatric studies of guidelines that used AGREE-II [15,16]. A scaled domain score of <60% was considered insufficient, >60% was sufficient quality and a score of >80% was good quality. To decide whether guidelines could be recommended, not recommended, or recommended with modifications based on the reviewer’s scores, we used the method described by Chiappini et al., where guidelines with an overall score of 6 or more were recommended, scores of ≥4 but <6 were recommended with modification, and scores of ≤3 were not recommended [15]. Modifications recommended to guidelines were specified according to the lowest-scoring domains. For example, including more information on editors and financial contributions to satisfy editorial independence could be considered a modification. ICC and 95% confidence intervals (CI) were calculated for each guideline and domain using mean-rating (k = 5), consistency, 2-way random-effects model [17]. 

**Table 1:** Characteristics of eligible clinical practice guidelines identified in systematic search.

Title of Guideline	Country/State of Publication *	Institute	Evidence- or Consensus-Based Grading System	Publication Year	Date for Next Guideline Review
National Clinical Guidelines for the management of substance use during pregnancy, birth and the postnatal period [18]	Australia/National	New South Wales (NSW) Ministry of Health	Evidence- and consensus-based—NHMRC	2014	Not specified
Perinatal substance use: neonatal [19]	Australia/Queensland	Queensland Health	Evidence-based ^	2021	September 2026
Neonatal Abstinence Syndrome Guidelines [20]	Australia/New South Wales	NSW Ministry of Health	Evidence-based ^	2013	September 2018 †
Neonatal Abstinence Syndrome (NAS) [21]	Australia/South Australia	SA Health	Evidence- and consensus-based ^	2022	February 2027
Substance use during pregnancy—care of the mother and newborn [22]	Australia/Victoria	Safer Care Victoria: Centre of Clinical Excellence	Evidence-based ^	2018	September 2020 †
Neonatal Abstinence Syndrome [23]	Australia/Western Australia (WA)	Government of Western Australia	Evidence-based ^	2020	February 2023
Managing infants born to mothers who have used opioids during pregnancy [24]	Canada	Canadian Paediatric Society (CPS)	Evidence-based ^	2018	Not specified
Neonatal abstinence syndrome clinical practice guidelines for Ontario [25]	Canada/Ontario	Provincial Council for Maternal and Child Health	Evidence-based—CTFPHC	2012	Not specified
Care of the Newborn Exposed to Substances During Pregnancy [26]	Canada/British Columbia	Perinatal Services BC	Evidence-based ^	2020	Not specified
Neonatal abstinence syndrome [27]	UK/Scotland	NHS (Greater Glasgow and Clyde)	Evidence-based ^	2019	February 2022 †
Joint Trust Guideline for the Management of Neonatal Abstinence Syndrome [28]	United Kingdom (UK)/England	NHS (Norfolk and Norwich)	Evidence-based ^	2021	July 2024
Neonatal Abstinence Guideline [29]	UK/England	NHS (North Devon)	Evidence-based ^	2019	July 2022 †
Thames Valley Guideline for Neonatal Abstinence Syndrome [30]	UK/England	NHS (Thames Valley and Wessex)	Evidence-based ^	2017	September 2019 †
Guideline on the Management of Neonatal Abstinence Syndrome [31]	UK/Wales	National Health Service (NHS) (Wales)	Evidence-based ^	2017	October 2020 †
Neonatal drug withdrawal [32]	United States of America (USA)	American Academy of Pediatrics (AAP) (Hudak)	Evidence-based ^	2012	May 2021 †
Neonatal Opioid Withdrawal Syndrome [33]	USA	AAP (Patrick)	Evidence-based ^	2020	November 2025
ABM clinical protocol #21: guidelines for breastfeeding and substance use or substance use disorder, revised 2015 [34]	USA	American Breastfeeding Medicine (ABM)	Evidence-based ^	2015	April 2020 †
Committee Opinion No. 711: Opioid Use and Opioid Use Disorder in Pregnancy [35]	USA	American College of Obstetricians and Gynecologists (ACOG)	Evidence-based ^	2017	May 2021—Renewed
Pregnancy and Opioid Exposure: Guidance for North Carolina [36]	USA/North Carolina	North Carolina Pregnancy and Opioid Exposure Project	Evidence-based ^	2015	Not specified
Clinical Guidance for Treating Pregnant and Parenting Women with Opioid Use Disorder and Their Infants [37]	USA	Substance Abuse and Mental Health Services Administration (SAMHSA)	Consensus-based	2018	Not specified
Management of Neonatal Opioid Withdrawal [38]	USA/Vermont	Vermont Department of Health	Evidence-based ^	2010	Not specified
Guidelines for the identification and management of substance use and substance use disorders in pregnancy [39]	International	World Health Organisation (WHO)	Evidence-based—GRADE system	2014	2019 †

An overview of guideline characteristics, according to country and state of publication if applicable, and publishing institution. Evidence-based was characterised by reference lists in guidelines or references to evidence-based processes in guideline methodology. Consensus-based was characterised by reference to a consensus process in guideline methodology. Year of publication and review date, as given by the guideline itself or institution-wide, was also provided, in addition to update progress. GRADE: Grading of Recommendations Assessment, Development and Evaluation; NHMRC: National Health and Medical Research Council; CTFPHC: Canadian Task Force on Preventative Health Care. * A state is defined as a nation or territory considered as an organised political community under one government. This is given as a ‘state’ in Australia and the United States, ‘province’ in Canada and ‘country’ in the United Kingdom. State is used to encompass all these terms for simplicity. † No updated guidelines are available after the specified review date. ^ No grading system provided.

**Table 2:** Overview of recommendations for treatment and follow-up within clinical practice guidelines.

Guidelines	Recommendations for Treatment								Recommendations for Follow-up	
	Antenatal Screening	Toxicology Screening at Birth	Assessment Tool	Breastfeeding if Mother is Stable on OMT and No Transmissible Viruses	Non-Pharmacological Management	Pharmacological Management with Oral Morphine Hydrochloride Dose *	Additional Pharmaceutical Agent as Second Line (e.g., Clonidine, Methadone, Phenobarbitone)	Outpatient Pharmacotherapy	Short-Term Follow-up with GP or Paediatrician (within Specific Time Frame)	Long-Term Follow-up with Development Assessments (within Specific Time Frame)
Australia/National [18]	+	+	+Finnegan	+	+	+	+	-	-	-
Australia/Queensland [19]	+	-	+Finnegan and Eat, Sleep, Console	+	+	+0.125 mg/kg/dose, 6 hourly	+	+	+	+
Australia/New South Wales [20]	+	+	+Finnegan	+	+	+0.125 mg/kg/dose, 6 hourly	+	+	+	-
Australia/South Australia [21]	+	+	+Finnegan	+	+	+ 0.1 mg/kg/dose, 4 hourly	+	+	+	-
Australia/Victoria [22]	+	+	+Finnegan	+	+	+ 0.125 mg/kg/dose, 6 hourly	+	+	+	-
Australia/Western Australia [23]	+	-	+Finnegan	+	+	+0.125 mg/kg/dose, 6 hourly	+	+	-	-
Canada/CPS [24]	+	+	+Finnegan	+	+	+ 0.05 mg/kg/dose, 6 hourly	+	+	-	-
Canada/Ontario [25]	+	+	+Finnegan	+	+	+ 0.05 mg/kg/dose, 6 hourly	+	+	-	-
Canada/Perinatal BC [26]	+	+	+Eat, Sleep, Console	+	-	+ 0.04 mg/kg/dose, 4 hourly	-	-	-	-
United Kingdom (UK)/Greater Glasgow and Clyde [27]	+	+	+Lipsitz	+	+	+ 0.06 mg/kg/dose, 4 hourly	+	-	-	-
United Kingdom (UK)/Norfolk and Norwich [28]	-	+	+Lipsitz	+	+	+ 0.05 mg/kg/dose, 4 hourly	+	+	-	-
United Kingdom (UK)/North Devon [29]	+	-	+Own system	+	+	+ 0.04 mg/kg/dose, 4 hourly	+	+	-	-
United Kingdom (UK)/Thames Valley and Wessex [30]	+	-	+Own system	+	+	+ 0.04 mg/kg/dose, 4 hourly	+	+	-	-
United Kingdom (UK)/Wales [31]	+	+	+Lipsitz	+	+	+ 0.06 mg/kg/dose, 4 hourly	+	-	-	-
USA/AAP (Hudak, 2012) [32]	+	-	+	+	+	+ 0.04 mg/kg/dose, 3–4 hourly	+	+	-	-
USA/AAP (Patrick, 2020) [33]	+		+	+	+	+	+	+	-	+
USA/ABM [34]	+		+	+	-	-	-	-	-	-
USA/ACOG [35]	+		+Finnegan	+	-	-	-	-	-	-
USA/North Carolina [36]	-		+Finnegan	+	+	+	-	+	+	-
USA/SAMHSA [37]	+		+	+	+	+	+	+	+	-
USA/Vermont [38]	-		+Finnegan	+	+	+ 0.04 mg, 3–4 hourly	-	+	+	-
International/WHO [39]	+		+	+	+	+	+	-	-	-

Overview of recommendations provided in the clinical practice guidelines according to antenatal care, assessment of NAS non-pharmacological and pharmacological management, and follow-up. Extra details regarding the scoring systems used and the opioid doses are provided for additional context. + Indicates that the recommendation was explicitly mentioned within the guideline. - Indicates that there was no explicit mention of the recommendation with the guideline. * Recommended initial dose regimen.

## 3. Results

In total, 1703 articles were identified based on title and abstract reviews with duplicates removed. A total of 1505 articles that did not meet the selection criteria were excluded, leaving 198 records for full-text review. After the full-text screening process, thirty-three CPGs were assessed for eligibility, with one record not retrieved as the website was unavailable. Among these, eleven articles were excluded, with six duplicates of CPGs already included for analysis [24,25,26], one conference paper [40], and four not specific to NAS clinical management (shown in Figure 1) [41,42,43,44]. The final analysis included twenty-two CPGs with seven from the USA, three from Canada, five from Australia, five from the UK and one international guideline. Notably, nine guidelines were out of date according to their own updating criteria and seven had no update date specified. Table 1 provides a general overview of the characteristics of the included CPGs.

### 3.1. Summary of Recommendations 

#### 3.1.1. Antenatal and Toxicology Screening

A total of 86% of guidelines recommended obtaining a maternal substance use history during antenatal care (Table 2). Specifically, they all recommended that clinicians should ensure that a drug use history be obtained from all women attending antenatal care at the initial assessment and that this should be repeated periodically throughout pregnancy to detect drug-use changes. A total of 59% of CPGs recommended toxicology screening of the infant at birth with either urine, meconium or hair samples to further determine substance exposure (Table 2). 

#### 3.1.2. Diagnosis 

All CPGs recommended the monitoring of infants at risk of neonatal withdrawal using a clinical assessment tool. Specifically, 68% of guidelines either included or directly referred to the Finnegan Score for the diagnosis of NAS. The remaining seven guidelines did not use the Finnegan Score. Two recommended an institutional-based tool [29,30], two recommended the Eat, Sleep, Console (ESC) instrument [19,26], and three recommended the Lipsitz tool [27,28,31]. 

#### 3.1.3. Management 

Supportive or non-pharmacological management (e.g., quiet environments, swaddling, breastfeeding) was recommended by all guidelines as a first-line treatment for babies with prenatal substance exposure. Pharmacotherapy was recommended if supportive therapy did not adequately control the symptoms of withdrawal. Opioids were recommended as first-line pharmacotherapy by 91% of guidelines, whilst 94% of guidelines recommended phenobarbitone as a second-line agent if symptoms were not controlled by opioids or if non-opioid withdrawal was suspected. A small proportion of guidelines also suggested alternative drugs such as methadone (9%) [28,32] as a first-line treatment and clonidine (27%) [18,22,23,32,33,37] as a second-line, but they did not provide dosing regimens for these. Most guidelines suggested that reductions in medication doses should only be considered when there had been 48–72 h of clinical stability as indicated by at least three consecutive Finnegan scores of <8 [45].

#### 3.1.4. Follow-Up

Although 59% of guidelines mentioned the need for follow-up of the infant after hospital discharge or after cessation of withdrawal, only 36% (*n* = 8) provided details regarding follow-up procedures, including recommended members of the multidisciplinary team and the frequency and duration of follow-up. For example, the CPG from the Queensland Government recommended that babies should receive a medical review within one week of discharge and that ongoing surveillance with a general practitioner and/or paediatrician, social worker and other community services was strongly encouraged. 

Table 2 provides a summary of the recommendations as a proportion of the total number of guidelines, and further outlines the specific recommendations.

### 3.2. Quality Appraisal and Inter-Rater Reliability

#### 3.2.1. Domain 1: Scope and Purpose

The domain of scope and purpose evaluates the degree to which the overall objectives, clinical questions and target audience of the guideline are specifically described. The median standardised score of the guidelines in the review was 72% (range: 33–94%). The CPG with highest score was from the WHO (94%) [39]. The lowest score (33%) was from Queensland Health [19]. 

#### 3.2.2. Domain 2: Stakeholder Involvement

Stakeholder involvement reflects the guideline’s ability to represent the views and preferences of key participants. The median standardised score of all CPGs was of insufficient quality (37%, range: 0–96%), with the highest score from the WHO (96%) [39] and lowest from Wales, UK (0%) [31].

#### 3.2.3. Domain 3: Rigour of Development

Rigour of development describes the methodological processes guiding CPG development, including systematic evidence searches, criteria for evidence selection and recommendations for the benefits and risks to health. The median score for this domain was 33% (range: 1–88%), i.e., insufficient quality, with highest score from the WHO (88%) [39]. 

#### 3.2.4. Domain 4: Clarity of Presentation

Clarity of presentation assesses whether key recommendations are specific, unambiguous and easily identifiable. This domain had the highest median score of 85% (range: 59–100%), with only the Wales, UK, guideline scoring < 60% with 59% [31]. WHO had the highest score of 100% [39].

#### 3.2.5. Domain 5: Applicability

This examines the ability of CPG to be implemented in everyday clinical practice, inspecting organisational barriers, cost implications and auditing criteria. The overall median standardised score was 34% (range: 11–67%). The Ontario CPG scored > 60% [25]. All others provided insufficient information to inform on translation and implementation strategies.

#### 3.2.6. Domain 6: Editorial Independence

Editorial independence evaluates the presence of conflicts of interest and whether the guideline is independent of its funding body. The median standardised score was 0% (range: 0–100%). The only guideline to fulfil all criteria was that from the American Academy of Pediatrics (AAP) in 2012 (100%) [32], followed by the WHO (73%) [39] and the AAP’s 2020 guideline (70%) [33]. 

### 3.3. Summary of Evaluation

Following evaluation using the AGREE-II instrument, the WHO CPG was the only one that could be recommended for clinical use without modification [39]. Both AAP [37,38], Australia [18], Ontario [25] and Substance Abuse and Mental Health Services Administration (SAMHSA) [37] CPGs could be recommended with modifications or updates. The remaining sixteen could not be recommended for clinical use. A tabulated summary of domain values can be seen in Table 3.

The ICC values for all guidelines were >0.902 (range: 0.902–0.988) (Table 4). When ICC was calculated per domain, the values were >0.926 (range: 0.926–0.983) (Table 5). Scores from 0.810 to 1.00 are indicative of a very good level of agreement [17].

## 4. Discussion 

Despite the increasing prevalence of NAS, there remains a paucity of evidence-based guidance for the assessment and management of this very vulnerable population. We identified only 22 CPGs from four countries: Australia, Canada, the UK and the USA. Most CPGs were outdated and none specified a revision timeframe. Not surprisingly, reviews of front-line clinical practice find variations of up to 100% between hospitals in their treatment of mothers and infants with a history of prenatal drug use [46]. 

These inconsistencies are likely fuelled by CPGs that are outdated and limited in their capacity to provide clear and specific guidance for clinicians. There are two possible explanations for this based on this study’s findings. Firstly, that the existing evidence base underpinning these CPGs is lacking and based on small cohort studies. When assessed against the current literature, the most consistent evidence-based recommendation was to obtain early and repeated antenatal histories of drug use. This, however, is a fraught assessment and very dependent on the clinician obtaining the history, the relationship between the patient and the clinician and the consequences of maternal disclosure of drug use [47]. For example, some states in the USA consider any disclosure of maternal drug use to be a criminal offence and this, not unexpectedly, drives down voluntary and accurate maternal disclosure of drug use [48]. The second explanation for unclear CPGs is that the guidelines are poorly written in fundamental domains, as indicated by the AGREE-II scores.

### 4.1. Recommendations for Clinical Diagnosis

Many guidelines recommended the Finnegan Neonatal Abstinence Scoring System (FNASS) as the assessment tool of choice to monitor and treat infants at risk of NAS. Indeed, this tool was used by >89.5% of participants from Canada, one of the primary countries publishing NAS CPGs, despite the tool’s significant limitations [49]. The FNASS was developed by Finnegan et al. in 1975 as a research tool to distinguish the severity of withdrawal and response to pharmacological treatment in infants with known narcotic exposure who were full- or near-term, bottle-fed and treated with medications that are not used today, including paregoric, diazepam and tincture of opium [45]. It, and other assessment tools, including the Eat, Sleep, Console (ESC) tool developed by Grossman et al. (2017) [50] have never been validated for non-opioid exposure or for impact on neurodevelopmental or longer-term outcomes, considering the potential neurotoxicity of many of the medications that are used to treat NAS [51]. Regarding new evidence for assessment tools, a recent randomised controlled trial from Young et al. (2023) evaluated the ESC, finding that length of stay was reduced from 14.9 to 8.2 days without increased incidence in adverse outcomes for up to three months’ post-discharge [5]. While ESC is still within its infancy, there is an indication that this could be a reliable and valid assessment tool with further studies. 

### 4.2. Recommendations for Management

Recommendations for non-pharmacological management, such as swaddling, low-stimuli environments and breastfeeding, were unanimous, although the evidence is low regarding the benefits for infant outcome. A systematic review and meta-analysis found that non-pharmacological measures contributed to only a one-day reduction in length of hospitalisation (95% CI −2.82–0.82) [52]. Breastfeeding, on the other hand, has been demonstrated to significantly reduce withdrawal severity and has decreased length of stay by as much as 3 weeks [53,54]. This could be due to the pacifying act of skin-to-skin contact and the transfer of small amounts maternal drug via breastmilk [55]. However, one of the most important contributing factors could be that mothers who are committed to breast feeding are more likely to be more stable in their drug use and more responsive to their infants’ needs, which also decreases withdrawal severity. 

In severe NAS that is unresponsive to non-pharmacological management, medication is life-saving [56]. However, not all hospitals have standardised medication protocols, especially non-tertiary hospitals [57], which increases the risk of medication complications. Most guidelines recommended morphine as the first-line pharmacological agent in known opioid-exposed infants even though morphine has not been approved by any country to treat newborn withdrawal [58]. However, there have been ongoing studies investigating dosing and treatment regimens for NAS with other medications such as buprenorphine [59], which may be a promising avenue for the future. 

Guidance for the appropriate use of second-line medications was even less robust, with recommendations varying between clonidine, phenobarbitone and other agents. Evidence for the use of adjunctive medications was limited to hospitalisation only. For example, a prospective randomised study of 25 infants found that treatment with phenobarbitone as an adjunct to morphine significantly reduced length of morphine treatment (26 days versus 34 days) and length of stay (31 days versus 42 days) when compared to the group treated with clonidine [60]. There is currently no proven optimal adjunctive drug to morphine for treating opioid-related NAS and no defined safest or optimum medication for treating infants with non-opioid withdrawal [61,62].

Almost no CPG provided a translatable and implementable pathway of care for after hospital discharge, including recommendations for child protection surveillance or neurodevelopmental follow-up and intervention. This is of great concern because parental drug use has one of the most important associations with child harm [63]. Longitudinal population studies suggest strongly that children with a history of NAS are significantly more likely to die and be hospitalised for preventable issues such as trauma, injury and abuse and experience serious cognitive deficits and poor neurological outcomes, including cerebral palsy [64]. 

### 4.3. Guideline Quality Appraisal

The quality of the CPGs was assessed using AGREE-II, with insufficient scores in the domains of rigour of methodology, applicability and editorial independence. Poor scores in these areas significantly impact the utility and value of CPGs as they are vital to the interpretation and implementation of recommendations. The rigour of methodology has been demonstrated to be a systematically weak domain in medical CPGs [65]. Time, resource and manpower constraints have been suggested as explanations for these limitations, with resulting impacts on the reliability of recommendations [66]. Applicability in the appraised CPGs was low, and this is of concern considering most NAS assessment tools, such as the FNASS, are subjective and require educational materials [50]. Finally, the domain of bias and conflict of interest scored poorly, which can significantly affect healthcare decisions if there are relationships between CPG publishers and pharmaceutical companies, governments or other industries [67]. Future CPGs should make a conscious effort to manage funding sources and biases, so that healthcare providers are able to make informed management decisions.

Based on the reviewer’s conclusions, only the WHO CPG was recommended for use [39], and five were recommended with modifications: Both AAP, Australian, Ontario and SAMHSA. Examples of modifications would include providing stakeholder input in the recommendations through either direct surveys or literature citation for the AAP and Ontario CPGs [25,32,33]. Methods for how the guidelines were formed needed to be included in greater detail in all five guidelines, such as signposting whether they were evidence- or consensus-based, as well as more specific advice for applicability. The Australian, Ontario and SAMHSA guidelines required modification of their editorial independence, including disclosing all funding sources, author affiliations and conflicts of interest if these were applicable [18,25,37].

This study had several strengths. Notably, it is one of the first studies to systematically identify and review CPGs for NAS. Efforts were made to ensure the search and guideline evaluation were as thorough as possible. These measures included producing a pre-specified protocol according to the PRISMA guidelines. Stringent inclusion and exclusion criteria were implemented and the thoroughly evaluated AGREE-II was used for quality appraisal. 

However, there are some limitations to this study. Despite the efforts made to ensure all CPGs on NAS management were accounted for, many internal hospital network CPGs are unpublished and it was not possible to obtain all CPGs that have ever been produced for NAS. The investigators had to define cut-offs for domain quality, as these are not provided with AGREE-II. This introduced a risk of assessor bias, an issue reflected in other studies using this instrument [15]. Attempts to address this included using a pilot scoring exercise, discussion between reviewers when differences arose and calculating reliability using an ICC statistic.

Finally, we acknowledge that the effect of any CPG declines with time in light of new evidence and research. However, we did not examine the effect of date of publication in our study because all the CPGs that we assessed were freely available, suggesting that they, despite considerable changes in NAS care over the last decade, are considered by policy makers and clinicians to be current recommendations for clinical practice.

## 5. Conclusions

This study highlights the predominance of low-quality recommendations in CPGs for NAS, impacting recommendations for assessment and management. This can be improved with both higher-quality scientific evidence and systematically written and designed CPGs. The increasing incidence of substance use in pregnancy and NAS diagnoses has a multifaceted impact on the economy, society and marginalised populations. The creation of well-designed studies and standardised, evidence-based CPGs is imperative to improve the outcomes of these infants and their families.

## Figures and Tables

**Table 3:** Standardised domain scores for each clinical practice guideline and overall recommendation (*n* = 22).

Country of Origin	Author	Scaled Score Percentage (%)						Overall Recommendation
		1. Scope and Purpose	2. Stakeholder Involvement	3. Rigour of Development	4. Clarity of Presentation	5. Applicability	6. Editorial Independence	
Australia	National [18]	81	78	51	94	57	30	Y with M
	Queensland Health [19]	33	72	42	96	26	16	N
	New South Wales Health [20]	72	37	37	63	32	0	N
	South Australia Government [21]	81	46	21	94	36	0	N
	Victorian Government [22]	57	1	13	78	11	0	N
	Western Australia Health [23]	39	30	28	65	31	0	N
Canada	CPS [24]	70	39	34	65	17	22	N
	Ontario [25]	87	24	45	94	67	0	Y with M
	Perinatal BC [26]	54	11	26	87	54	0	N
United Kingdom	NHS/Greater Glasgow and Clyde [27]	59	52	17	85	18	0	N
	NHS/Norfolk and Norwich [28]	48	56	38	70	31	0	N
	NHS/North Devon [29]	85	13	24	70	40	0	N
	NHS/Thames Valley and Wessex [30]	91	44	27	94	35	0	N
	NHS/Wales [31]	43	0	1	59	19	0	N
United States of America	AAP (Hudak, 2012) [32]	80	24	40	93	17	70	Y with M
	AAP (Patrick, 2020) [33]	91	17	38	96	36	100	Y with M
	ABM [34]	70	20	41	85	15	24	N
	ACOG [35]	74	37	31	78	32	0	N
	North Carolina [36]	91	17	20	72	36	0	N
	SAMHSA [37]	72	63	56	98	43	19	Y with M
	Vermont [38]	63	39	12	78	49	35	N
International	WHO [39]	94	96	88	100	43	73	Y
**Median Domain score ± SD**		72 ± 18	37 ± 25	33 ± 18	85 ± 13	34 ± 15	0 ± 29	

The Appraisal of Guidelines for Research and Evaluation Instrument (AGREE-II) was used to evaluate the quality of clinical practice guidelines by domain. The standardised score considers the scores of 3 independent reviewers. A score of <60% was an insufficient score, >60% was a sufficient score, and >80% was a good-quality score. Y: recommended for clinical use; M: modifications or updates to recommendations needed before clinical use; N: not recommended for clinical use; NHS: National Health Service; AAP: American Academy of Pediatrics; SAMHSA: Substance Abuse and Mental Health Services Administration; WHO: World Health Organisation; SD: standard deviation.

**Table 4:** Intraclass correlation coefficients and their confidence intervals per clinical practice guideline.

Guideline	ICC	95% CI
Australia		
National [18]	0.975	0.950–0.989
Queensland [19]	0.977	0.954–0.990
New South Wales [20]	0.981	0.961–0.991
South Australia [21]	0.964	0.927–0.983
Victoria [22]	0.968	0.935–0.985
Western Australia [23]	0.955	0.910–0.979
Canada		
CPS [24]	0.902	0.805–0.955
Ontario [25]	0.982	0.964–0.992
British Columbia [26]	0.982	0.965–0.992
United Kingdom		
Greater Glasgow and Clyde [27]	0.972	0.959–0.991
Norfolk and Norwich [28]	0.918	0.836–0.963
North Devon [29]	0.981	0.962–0.991
Thames Valley and Wessex [30]	0.952	0.904–0.978
Wales [31]	0.956	0.913–0.980
United States of America		
AAP (Hudak, 2012) [32]	0.973	0.946–0.988
AAP (Patrick, 2020) [33]	0.974	0.947–0.988
ABM [34]	0.943	0.886–0.974
ACOG [35]	0.950	0.900–0.977
North Carolina [36]	0.984	0.967–0.992
SAMHSA [37]	0.984	0.967–0.992
Vermont [38]	0.929	0.858–0.968
International		
World Health Organisation [39]	0.988	0.976–0.995

Intraclass correlation coefficients and 95% confidence intervals were calculated per guideline according to a mean-rating (k = 5), consistency, 2-way random-effects model. Scores from 0.810 to 1.00 are indicative of a very good level of agreement [17]. ICC—intraclass correlation coefficient. CI—confidence interval.

**Table 5:** Intraclass correlation coefficients and their confidence intervals per domain.

Domain	ICC	95% CI
1	0.947	0.920–0.966
2	0.974	0.961–0.983
3	0.958	0.946–0.968
4	0.926	0.889–0.953
5	0.940	0.915–0.959
6	0.983	0.972–0.990

Intraclass correlation coefficients and 95% confidence intervals were calculated per domain according to a mean-rating (k = 5), consistency, 2-way random-effects model. Scores from 0.810 to 1.00 are indicative of a very good level of agreement [17]. ICC—intraclass correlation coefficient. CI—confidence interval.

## Data Availability

The data presented in this study are available in this article, A Systematic Review of Clinical Practice Guidelines for Neonatal Abstinence Syndrome.
